# Perspective on sequence evolution of microsatellite locus (CCG)_n _in Rv0050 gene from *Mycobacterium tuberculosis*

**DOI:** 10.1186/1471-2148-11-247

**Published:** 2011-08-31

**Authors:** Lianhua Qin, Jie Wang, Ruijuan Zheng, Junmei Lu, Hua Yang, Zhonghua Liu, Zhenling Cui, Ruiliang Jin, Yonghong Feng, Zhongyi Hu

**Affiliations:** 1 Shanghai Key Laboratory of Tuberculosis, Shanghai Pulmonary Hospital, Tongji University School of Medicine, 507 Zhengmin Road, Shanghai, 200433, China

## Abstract

**Background:**

The mycobacterial genome is inclined to polymerase slippage and a high mutation rate in microsatellite regions due to high GC content and absence of a mismatch repair system. However, the exact molecular mechanisms underlying microsatellite variation have not been fully elucidated. Here, we investigated mutation events in the hyper-variable trinucleotide microsatellite locus MML0050 located in the Rv0050 gene of W-Beijing and non-W-Beijing *Mycobacterium tuberculosis *strains in order to gain insight into the genomic structure and activity of repeated regions.

**Results:**

Size analysis indicated the presence of five alleles that differed in length by three base pairs. Moreover, nucleotide gains occurred more frequently than loses in this trinucleotide microsatellite. Mutation frequency was not completely related with the total length, though the relative frequency in the longest allele was remarkably higher than that in the shortest. Sequence analysis was able to detect seven alleles and revealed that point mutations enhanced the level of locus variation. Introduction of an interruptive motif correlated with the total allele length and genetic lineage, rather than the length of the longest stretch of perfect repeats. Finally, the level of locus variation was drastically different between the two genetic lineages.

**Conclusion:**

The Rv0050 locus encodes the bifunctional penicillin-binding protein ponA1 and is essential to mycobacterial survival. Our investigations of this particularly dynamic genomic region provide insights into the overall mode of microsatellite evolution. Specifically, replication slippage was implicated in the mutational process of this microsatellite and a sequence-based genetic analysis was necessary to determine that point mutation events acted to maintain microsatellite size integrity while providing genomic diversity.

## Background

Simple sequence repeats (SSRs, also known as microsatellites) are repetitive sequence motifs of one to six base pairs (bps) scattered throughout all known genomes [1]. The extensive length differences that may be achieved by microsatellites and their high rate of polymorphism have facilitated their use as molecular markers in epidemiological investigations. However, knowledge is lacking about the mutational mechanism(s) that lead to variations in microsatellite loci.

Gaining a detailed understanding of the features underlying microsatellite genomic structure will aid subsequent interpretations of data from these clinically useful genomic regions. Strand slippage during DNA replication can cause insertion and deletion of repeat units in newly-synthesized nucleotide chains. These events are the most common cause of expansion or contraction of microsatellites [2,3]. Recent studies have reported differences in rates and patterns of mutation among distinct loci and species; thus, allele size, motif size, genetic lineage, G/C content, functional potential of the transcribed product, and effectiveness of mismatch repair enzymes might all act as mediators of the mutation patterns of such loci [4-7].

Although several models have been proposed to explain the mutation processes that effect microsatellite evolution, they have yet to be confirmed [6]. Studies into genomic evolution exploit the hyper-variable nature of microsatellite sequences to observe mutation events directly [8]. Specifically, pedigree analysis has provided substantial amounts of mutation data for broad ranges of chromosomal loci and organisms [8-10]. However, most of these studies on microsatellites have focused on size variation among alleles, and have not addressed potential sequence variations within otherwise similarly sized alleles.

Sequence analysis is an alternative empirical approach for studying microsatellite evolution. Elucidating the sequence structure of alleles allows for direct comparison with other alleles within a single species or with orthologous loci from different species, effectively allowing for the study of accumulated mutational effects over evolutionary timescales. Intraspecific comparisons that reveal the sequence structure of individual alleles may provide significant insights into the otherwise complex process of maintaining genomic integrity under selective pressure.

Mycobacterial genomes harbor a number of polymorphic microsatellites [11]. Microsatellites in these genomes impart a certain degree of genome plasticity and probably account for many biological functions in the context of pathogen adaptability, virulence and survival [12]. Usually, errors resulting from strand slippage are promptly repaired by a three-enzyme system composed of mutL, mutS, and mutH; however, mycobacterial genomes lack these enzymes [13]. Thus, such genomes serve as interesting systems to investigate the rates of mutations in microsatellites and the existence of regulatory mechanisms that govern microsatellite mutations.

The repetitive CCG sequence located in the Rv0050 gene in *Mycobacterium tuberculosis *(MTB) is a trinucleotide microsatellite locus (MML0050) that exhibits high a polymorphism rate. The Rv0050 locus encodes a bifunctional penicillin-binding protein (ponA1) [14,15]. The hyper-variable locus has gained popularity as a Variable Number Tandem Repeat (VNTR) biomarker in epidemiological investigations of MTB strains [16]. However, the mutational mechanisms that are responsible for generating the high levels of variation in the MML0050 locus remain unclear.

In this study, we sought to explain the mutation tempo and mode of the MML0050 locus and its polymorphic nature using clinical isolates from two MTB genotypes: W-Beijing and non-W-Beijing. W-Beijing strains are genetically closely related, present with a characteristic spoligotype pattern, and have enjoyed wide global dispersion [17-19]. They account for 80-90% of the MTB strains isolated from the Beijing area since the 1950s and remain prevalent in other parts of China, including the Ningxia, Shanghai, and Guangdong provinces [20]. Such strains have attracted much research interest due to their reported association with multiple drug resistance, relapses, treatment failure, hypervirulent phenotype in mice, and faster growth rates in human monocytes [21-25]. We sequenced a set of MML0050 locus alleles of different size classes from different MTB families to analyze the stabilizing effect of interrupting motifs in microsatellite regions, the effect of allele length, genetic lineage for the introduction of interruptive motif, and the relation between number of repeat units and mutation frequency.

## Methods

### Sampling and DNA extraction

The MTB reference strain H37Rv was studied along with 461 clinical strains that had been isolated between July 2009 and April 2010 from regions of Eastern China, including Shanghai, Jiangsu, Zhejiang, Shandong, Fujian, Anhui, and Jiangxi (collected by Laboratory of Tuberculosis, Shanghai Pulmonary Hospital, Medical School, Tongji University, Shanghai, China).

Strains were grown in Sauton culture medium (0.5 g/L KH_2_PO_4_, 0.5 g/L MgSO_4_.7H_2_O, 2 g/L citric acid, 0.05 g/L ferric ammonium citrate, 4.0 g/L L-asparagine, 6% glycerol and 0.02% Tween 80). Cells were sterilized at 80°C for 30 min, and were harvested by centrifugation (12,000 × g for 5 min). The bacterial pellet was washed three times with sterilized saline and re-collected by centrifugation (12,000 × g for 10 min each time). DNA Lysis Buffer (10 mmol/L NaCl, 1 mg/mL SDS, 15% Chelex-100, 1% Tween 20) was added and cells were incubated at 50°C for 1 h, followed by 100°C for 10 min. The mixture was centrifuged (5,000 × g for 10 min) to obtain the aqueous phase containing genomic DNA that was used for PCR amplification.

### Identification of W-Beijing strains

W-Beijing strains were identified by deletion-targeted multiplex PCR (DTM-PCR) to detect the genomic deletion RD105, which defines the W-Beijing family as a separate lineage within MTB [26-28]. The DTM-PCR primers P1 (5'-GGAGTCGTTGAGGGTGTTCATCAGCTCAGTC-3') and P2 (5'-CGCCAAGGCCGCATAGTCACGGTCG-3') were designed to amplify a 1466 bp product from the non-W-Beijing strains, while P1 and P3 (5'- GGTTGCCCACTGGTCGATATGGTGGACTT-3') were designed to amplify a 761 bp fragment from the W-Beijing genotype. PCR was performed in a 20 μL reaction mixture containing 2× Long *Taq *mixture (Tiangen, Co., Beijing, China), 0.2 μM of each primer and 10 ng of template DNA, under the following conditions: denaturation at 94°C for 5 min; 30 amplification cycles of 94°C for 30 s, 68°C for 30 s and 72°C for 2 min; and a final extension at 72°C for 7 min. PCR products were separated on 0.8% agarose gels.

### Microsatellite typing

To detect polymorphisms within the MML0050 locus, PCR primer pairs were designed for the flanking sequences: HponAF (5'-TTGAAGGGCACGTCGAACGAG-3') and HponAR (5'-GGGACCGATCGGGATGGTAA-3').

PCR was performed in a 20 μL reaction mixture containing 2× *Taq *mixture (Tiangen), 0.2 μM of each primer and 10 ng of template DNA, under the following conditions: denaturation at 94°C for 5 min; 35 amplification cycles of 94°C for 30 s, 65°C for 30 s and 72°C for 2 min; and a final extension at 72°C for 7 min. PCR products were analyzed by the PCR-Single Strand Conformation Polymorphism (SSCP) technique to detect point mutations; briefly, DNA was separated on a 6% polyacrylamide gel, visualized by silver staining, excised and sequenced on an ABI 3730xl DNA Analyzer (procedure carried out by Sangon, Shanghai, China).

GraphPad Prism 5.0 statistical software package (http://www.graphpad.com/prism/prism.htm) was used to analyze differences in allele distributions detected among the W-Beijing and non-W-Beijing genotypes. The character of bias was analyzed by Student's *t*-test with statistical confidence limit of 95%. Allele sequences were compared to sequences previously deposited in the GenBank database (http://www.ncbi.nlm.nih.gov/BLAST/). Phylogenetic trees were constructed by the neighbor-joining method using MEGA 4.0 software to ascertain the evolutionary distance of the locus mutation rates [29].

## Results and Discussion

### MML0050 microsatellite features revealed by fragment length analysis

**Figure 1:**
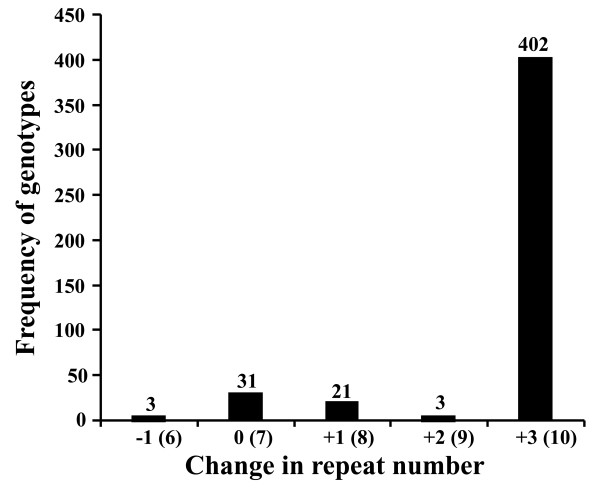
**Distribution of the MML0050 alleles in 462 *M. tuberculosis *strains as determined by PCR-SSCP**. The numbers in brackets refer to the repeat number of each allele.

The results from fragment length analysis indicated that the MML0050 locus in 462 strains comprised five alleles (Figure 1); this finding is consistent with molecular marker results reported by others [16]. The detected mutations involved gains or losses of either three, six, or nine bps. These trinucleotide units are equivalent to one, two, or three repeats, respectively, suggesting that replication slippage is associated with the mutation events. The distribution of alleles among the 461 clinical strains was evaluated next. Only three strains (0.65%) had contracting mutations, while 428 (92.8%) strains had expanding mutations; the remaining 30 strains presented with allele size identical to the H37Rv reference strain (Figure 1). Further analysis indicated that the relative frequency of alleles with -1, 0, +1, +2 and +3 change repeats were 0.65% (3/461), 6.5% (30/461), 4.55% (21/461), 0.65% (3/461), and 87.6% (404/461), respectively. The relative frequency of alleles with +2 change repeats (0.65%) was remarkably lower than alleles with the highest repeat numbers (87.6%), and even lower than alleles with 0 (6.5%) or +1 change repeats (4.55%). It appears that the mutation frequency of alleles did not correlate perfectly with an increasing number of repeat units (P = 0.0057, 95% CI: 0.2987 to 1.1300, *t *= 4.205). Thus, this finding suggests that repeat length is not the sole or primary mediator of mutational events in this MTB microsatellite.

**Figure 2:**
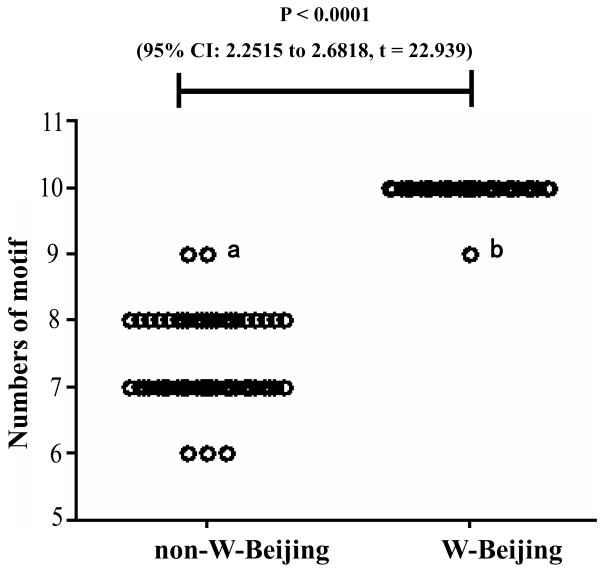
**Distributions of alleles from the MML0050 locus in W-Beijing and non-W-Beijing strains**. a: Rv0050*05 (CCG)_8_TCG; b: Rv0050*06 (CCG)_3_CCT (CCG)_4 _TCG.

Of the 461 clinical strains examined, 56 were defined as non-W-Beijing, similar to the H37Rv reference strain. The remaining 405 clinical isolates were defined as W-Beijing; finding that the majority (87.7%) of our samples were of this genotype was not surprising since W-Beijing is highly prevalent in Eastern China [21]. When allele size was examined (W-Beijing *vs*. non-W-Beijing) the comparison revealed that the levels of the locus variation were significantly different between the two groups (P < 0.0001, 95% CI: 2.2515 to 2.6818, *t *= 22.939; Figure 2). We found that 55 of the non-W-Beijing strains contained five alleles with -1, 0, +1, or +2 change repeats, while all of the W-Beijing strains contained 2 alleles with +2 or +3 change repeats. Moreover, in the W-Beijing group, nearly all samples (> 99%) harbored the allele with the highest repeat numbers. As such, this locus was determined to not represent a good molecular marker for epidemiological investigations of the W-Beijing genotype; it may still be a useful VNTR marker for other MTB strains, however, due to its high rate of polymorphism [16].

### MML0050 microsatellite features revealed by sequence analyses

We selected 132 strains for sequence analysis using the HponAF and HponAR primers. This group included 58 strains with -1, 0, +1, or +2 change repeats, and 71 strains randomly selected from among the 404 strains with +3 change repeats. Seven alleles at the MML0050 locus were identified by sequence analysis: Rv0050*01, Rv0050*02, Rv0050*03, Rv0050*04, Rv0050*05, Rv0050*06, and Rv0050*07 (Table 1). Comparison of the allele sequences to those in the GenBank database revealed that all, except Rv0050*05, matched to the *M. tuberculosis *complex (MTC) strains. The MTC group is comprised of Mycobacterium species targeting a broad range of hosts but having highly conserved sequences, including *M. tuberculosis, M. bovis, M. bovis *Bacillus Calmette-Guérin (BCG), *M. africanum*, and *M. microti*.

**Table 1:** Alleles of MML0050 locus in the Rv0050 gene from *M. tuberculosis*

Alleles	Sequences	Mutation patterns			Strains
			
		Insertion	Deletion	Substitution	
Rv0050*01	------------------CCGCCGCCGCCGCCGCCGCCG				H37Rv
Rv0050*02	------------------CCGCCGCCGCCGCCGCCGTCG			C→T	H37Ra, CDC1551, KZN1435
Rv0050*03	------------------------CCGCCGCCGCCGCCGTCG		1 motif	C→T	clone Y219
Rv0050*04	------------CCGCCGCCGCCGCCGCCGCCGTCG	1 motif		C→T	F11
Rv0050*05	------CCGCCGCCGCCGCCGCCGCCGCCGTCG	2 motifs		C→T	
Rv0050*06	------CCGCCGCCGCCTCCGCCGCCGCCGTCG	2 motifs		G→T; C→T	*M. Bovis*
Rv0050*07	CCGCCGCCGCCGCCTCCGCCGCCGCCGTCG	3 motifs		G→T; C→T	*M. Bovis *BCG

Variations in the genome may occur by other modes besides insertion/deletion. Nucleotide substitutions, such as transitions (A→G) or transversions (G→T), may alter the genome and its function without affecting the structure itself. Sequence analysis revealed that the Rv0050*01 allele was composed of seven perfect CCG repeats from the MTB reference strains H37Rv. All alleles from the clinical strains had C→T substitutions in the last repeat motif. Rv0050*07 was composed of 10 repeat units and had the largest fragment size; moreover, this allele had G→T substitutions in the fifth repeat motif, which interrupted the CCG consecutive repeat.

Microsatellite alleles can generally be divided into two types: alleles that are identical in both length and sequence and those identical in length but not in sequence, the latter being known as homoplastic microsatellite alleles. Rv0050*05 and Rv0050*06 had been determined by fragment length analyses to be identical in length (both having nine repeat units) but sequence analysis revealed significant differences in the underlying genomic sequences. Rv0050*05 had a perfect repeat with eight CCG units, while Rv0050*06 had a G→T substitution in the fourth repeat motif and the same mutation pattern of Rv0050*07. Overall, only 7.4% of the MML0050 mutations detected in our samples resulted in homoplastic character.

**Figure 3:**
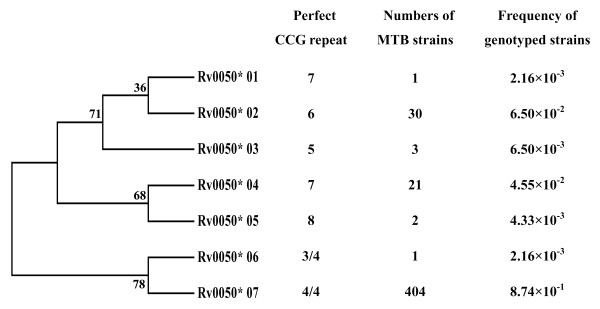
**The neighbor-joining tree of MML0050 alleles based upon the aligned sequence using MEGA4.0 software**.

A phylogenetic tree was constructed using the seven MML0050 alleles to determine evolutionary distance of the sequences (Figure 3). MML0050 alleles clustered into two major groups according to genotype: non-W-Beijing (Rv0050*01, Rv0050*02, Rv0050*03, Rv0050*04, and Rv0050*05) and W-Beijing (Rv0050*06 and Rv0050*07). The non-W-Beijing genotype group was further divided among three subtypes: Rv0050*01 and Rv0050*02, Rv0050*03, and Rv0050*04 and Rv0050*05. Although the Rv0050*05 and Rv0050*06 were identical in length, they were assigned to different clusters according to the point mutation sequence differences (Figure 2). Our finding of such homoplastic alleles was directly facilitated by the use of a nucleotide sequence-based genetic technique; genetic approaches lacking sequence data may hide polymorphisms, such as those from point mutation, and negatively impact the usefulness of this genetic marker in population genetics studies. Polymorphism of molecular markers should be calculated not only based on size variations but also the sequence difference.

We also analyzed the relation between number of interruptive motifs and (1) the total length of allele or (2) genetic lineage. The Rv0050*06 allele from W-Beijing genotype with nine repeat motifs was determined to be the point of introduction of a second interruptive motif. In contrast, the Rv0050*05 allele belonging to the non-W-Beijing genotype had a perfect nine repeat. Alleles from the non-W-Beijing genotype had a greater amount of perfect repeat motifs than did those from the W-Beijing genotype. Introduction of an interruptive motif had the strongest statistical correlations with the total length of allele and genetic lineage (P < 0.0001, 95% CI: 6.482 to 8.946, *t *= 13.64). Surprisingly, the length of the longest stretch of perfectly repeated units was not associated with any interruptive motif, suggesting the existence of a threshold level for the maximum length of perfectly repeated trinucleotide motifs in stable MML0050 alleles. In addition, this finding suggests that genetic lineage and point mutation may be moderators of mutational events in those alleles with nine repeat motifs.

### Evolution of the MML0050 locus

**Figure 4:**
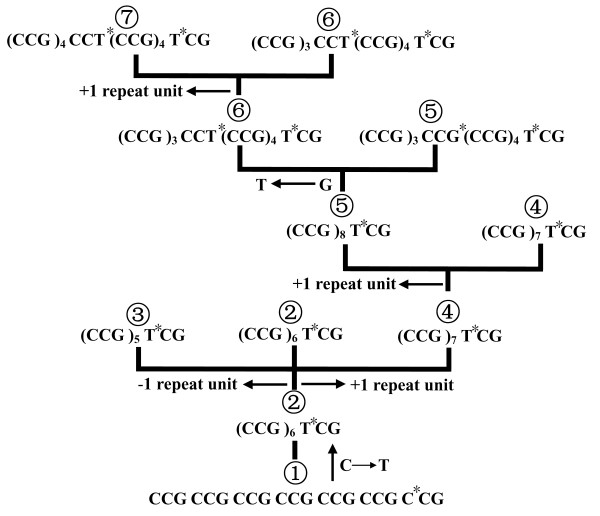
**Mutation models of trinucleotide microsatellite locus MML0050 located in the Rv0050 gene**. *Location of base substitution.

To investigate the evolution of the MML0050 locus we first considered the observed C→T substitution mutation that occurred in the last repeat motif and which differentiated all the clinical strains from the reference strain. This 100% mutation frequency may indicate that this nucleotide transition is a fixed mutation in MTB and one that has essentially benefited the strain's survival and/or host infectivity. Moreover, detailed investigation of the mutations in the alleles indicated that they occurred via a stepwise mutation process (SSM) of gaining or losing a motif until the repeat number reached as high as nine, at which point a G→T point mutation occurred to break long repeat arrays into smaller units (Figure 4). Directionality in the mutation process in favor of gains over losses has been observed in many eukaryotic genomes [7,30], while mutations from prokaryotic genomes show the opposite bias towards losses [30]. Our data on the MML0050 locus demonstrated that sequence gains (expansion) were much more prevalent than loses (contractions). The MML0050 allele sizes in individual isolates were very similar and often only differed by three base pairs, indicating that replication slippage is a likely mechanism underlying size-related polymorphisms in the MML0050 locus.

Undoubtedly, the high GC content (65.6% in MTB) [31] and absence of mismatch repair system (MMR) make the mycobacterial genome prone to polymerase slippage [13] and lead to a high mutation rate in simple sequence repeats [32]. However, the hyper-variable trinucleotide microsatellite locus MML0050 locus does not contain conspicuously long simple sequence repeats. Our analysis of W-Beijing and non-W-Beijing strains suggested that 10 repeats represented the upper limit for allele size in the MTB genome. Wanner *et al*. proposed context-dependent codon choice as an alternative mechanism used by the bacteria to reduce the number of mutations [32]; however, this mechanism does not fit with trinucleotide-related events. It has also been suggested by several others that infinite microsatellite growth may be disabled by introductions of point mutations [6,8,33]. Specifically, if the balance between slippage and point mutation favors point mutations within the repeated region, the mutations may interrupt the feature of the microsatellite without eliciting large changes in length. Indeed, all of the clinical strains examined in our study contained point mutations. This finding has led us to speculate that the first point mutation in the last repeat motif of all sequences may represent one of the bacteria's mechanisms to confine the expansion or contraction of microsatellite sequences. In addition, the second point mutation in the middle of the sequence may act as an alternative way to maintain the stabilization of long alleles by breaking repeat arrays into smaller units.

Trinucleotide repeating sequences from the MML0050 locus are transcribed into a repeating series of prolines in the Rv005 protein sequence. It has been reported that repeating sequences of amino acids can affect the physical and chemical properties of proteins, and harbor the potential for producing gradual and predictable changes in protein action [34,35]. Penicillin-binding protein encoded by Rv0050 is not only a key cell wall synthesizing enzyme [14,15] but also plays an important role in regulating cell wall hydrolysis [36]. Depletion of the penicillin-binding protein results in misshapen bacterial cells and impaired growth [15,36]. However, it has been unclear whether repeating sequences of amino acids caused by the MML0050 locus can affect the protein function or bacterial phenotype and growth. Nevertheless, the results from our study indicated that nearly all samples in the W-Beijing group with unique phenotypes harbored the allele with the highest repeat numbers. It is possible that polymorphism of the MML0050 locus, to some extent, can affect functional properties of the proteins and may, subsequently, impact bacterial phenotype.

## Conclusion

Size analysis indicated the presence of five MML0050 alleles that differed in length by three base pairs, implicating replication slippage in the mutational process of this microsatellite. Although the relative frequency in the longest allele was remarkably higher than that in the shortest for all of the tested strains, mutation frequency was not completely related with the total length in this trinucleotide microsatellite. Moreover, nucleotide gains were found to have occurred more frequently than loses. Sequence analysis supported the notion that point mutation events acted to maintain microsatellite size integrity while providing genomic diversity. Introduction of an interruptive motif correlated with the total allele length and genetic lineage, rather than the length of the longest stretch of perfect repeats. Finally, the comparison of W-Beijing vs. non-W-Beijing strains revealed that the levels of the locus variation were significantly different between the two groups.

## Authors' contributions

LHQ conceived of the study and participated in the design of the study, performed the phylogenetic and statistical analyses, and drafted the manuscript. JW carried out the microsatellite typing studies. RJZ carried out the identification of W-Beijing strains. JML performed the collection and culture of clinical strains. HY performed sequence alignment and collection of the sequence data. ZHL participated in the microsatellite typing and performed the collection of the typing data. ZLC participated in the collection of clinical strains and helped to draft the manuscript. RLJ participated in the phylogenetic and statistical analyses. YHF participated in the design of the study and helped to draft the manuscript. ZYH performed the design of the study and coordination and helped to draft the manuscript. All authors read and approved the final manuscript.
